# Annotations

**Published:** 1901-07-20

**Authors:** 


### ANNOTATIONS.

### Brompton Hospital and Open-air Treatment.

It is satisfactory to find that the Brompton Hospital, in putting itself in line with recent developments in regard to the treatment of consumption, has determined to keep in its own hands the management of the convalescent branch which is to be established on the Chobham Ridges, and thus to break away from a tendency which is making itself manifest in various quarters to regard the "open-air method" as something peculiar and apart from the general treatment of the disease. Broadly it may be said that all "systems" of cure are bad, and there can be no doubt that directly systems become so crystallised into form and routine that the patient decides under what method he will place himself, the door to quackery is opened very wide indeed. That the patient should decide upon his own treatment, as he so often wants to do, is bad enough, but the evil does not end here, for the natural consequence is that rival "cures" and competing institutions are driven to advertise themselves in such terms as will appeal to an ignorant public, much to the detriment of medical progress, not to mention the cause of truth. A mother decides that her daughter shall undergo the "Weir-Mitchell treatment," and she appeals to her doctor, not for his advice, but merely that he may put her on the track as to where it can be done; people no longer consult their doctor as to how to get rid of their rheumatism, but rather as to whether their hearts are strong enough to bear such and such a "treatment" which they want to undergo. Lupus cases tell us at once that they don't want to be scraped; all they want to know is where they can get the "light treatment." As for *poitrinaires* they are the most knowing of the lot; their heads are full of minute differences between cures carried on at different institutions, and of the comparative virtues of "high altitudes," "open-air," and "over-feeding," and all they ask for is an introduction to the head of the establishment they have selected. It is often impossible to induce these good people to exercise common sense and reasonable self-control at home, but they would stand on their heads and eat saw-dust if it were part of a "treatment" in the virtues of which they had become convinced by the perusal of some ingeniously written pamphlet. Under these circumstances we sadly want someone to reassert what we should have thought to be the fairly obvious verity, that the physician ought to be the responsible person in all that concerns the treatment of the sick, and that it is his proper work, and indeed his duty, to adjust the details of the treatment to the peculiarities of each case, just as he would the ingredients and the doses of his prescription. It is satisfactory then to find that the authorities at the Brompton Hospital instead of setting up an establishment for the carrying out of a particular "cure" have decided upon building a country hospital at which the physicians may be enabled to carry out such treatment as they may find necessary for their patients, including, of course, that by "open-air."

### Sanitary Certificates and Seaside Lodgings.

As August comes near the thoughts of paterfamilias turn not unnaturally to the serious question of seaside lodgings—not an altogether pleasant subject for contemplation. It is by no means an easy task to find lodgings which will at the same time fit one's purse and please one's family. But if one endeavours to discover rooms which will not only combine these advantages, but will also give some sort of assurance as to their sanitary condition, the difficulty is much increased. It certainly would seem desirable that those who go to the seaside for health should take up their abode in a place at least as healthy as the house they left behind them. Yet what do they do? Too often they go into a house of which they know absolutely nothing. To the majority a smiling landlady and some clean linen seem quite proof enough that all is right, while the more exacting and inquisitive, for all their cleverness, are apt to be taken in by the presence of some patent "disinfector" conspicuously placed in the sanitary department. We do not see how this great and serious difficulty is to be got over unless the sanitary authorities in watering-places will generally adopt the plan, which some of them have found so useful, of undertaking the duty of inspecting houses and issuing certificates in proper form, testifying to their healthiness so far as drainage, water supply, and sanitary fittings are concerned. We think, however, that they might go further, and devise some means of preventing the letting of lodgings which have recently been occupied by infectious cases or by people suffering from offensive diseases. The anxieties of paterfamilias might thus be greatly lessened. We would suggest, however, that some plan should be adopted by which all these certificates should remain the property of the sanitary authorities and should be recalled by them when out of date. We have seen various faded certificates, of early date, still hanging up in seaside lodgings long after they have ceased to be of any meaning. It may be true that if these documents are examined a date will be found upon them, and that in the body of the certificate a limit is fixed to their validity; still Mr. Paterfamilias is a careless and a trustful person, especially when he is tired with his journey and wants his tea, and we think it would be better that the certificate should vanish when it ceases to certify and can do nothing but delude.

### The University of London.

The interesting ceremony which recently took place when the first Degree Day of the Birmingham University was celebrated ought to remind the people of London of their own miserable backwardness in university affairs. Mr. Chamberlain very properly described the functions of a university as being the imparting, testing, increasing, and applying of knowledge. In all these departments the Birmingham University is already fully at work after only fifteen months of existence, while poor London, notwithstanding the Act passed in 1898 for the reconstruction of the so-called University of London, still remains apparently as far off as ever from being provided with a practical teaching university such as Birmingham has supplied itself with. The incubus of the old university, which never was in any true sense of the term a university for London students, still lies heavily upon all efforts to provide for Londoners what every provincial town of any size appears able to establish for the benefit of its inhabitants. The Long Vacation will soon be upon us, yet there are no signs of any decision having been arrived at by the University of London as to the admission of internal students under such conditions as shall enable them to obtain a pass degree on terms somewhat similar to those in force at the other universities. So long as London remains unable to offer a degree to its average student as a reward for work in its medical schools, so long shall we see the humiliating spectacle of the sons of Londoners going to provincial towns for their professional education.

